# Digital Vaccine Initiative for Inflammatory Bowel Disease Patients Using the Consolidated Framework for Implementation Research

**DOI:** 10.1016/j.gastha.2026.101094

**Published:** 2026-08-12

**Authors:** George Salem, Bharati Kochar, Patricia Jones, Gael Haddad, Sandra Quezada

**Affiliations:** 1 Crohn's and Colitis Center at OU Health, Section of Digestive Diseases and Nutrition, Department of Medicine, University of Oklahoma Health Sciences Center, Oklahoma City, Oklahoma; 2 Division of Gastroenterology, Massachusetts General Hospital, Boston, Massachusetts; 3 Division of Digestive Health and Liver Diseases, University of Miami School of Medicine and Jackson Memorial Health System, Miami, Florida; 4 Faculty of Medicine, University of Aleppo, Aleppo, Syria; 5 Division of Gastroenterology and Hepatology, University of Maryland School of Medicine, Baltimore, Maryland

**Keywords:** Inflammatory Bowel Disease, Vaccination, Digital Health, Implementation Science, Patient Engagement

## Abstract

**Background and Aims:**

Digital health interventions may improve preventive care for patients with inflammatory bowel disease, but implementation barriers can limit effectiveness. We evaluated a multisite digital vaccine outreach initiative and identified implementation barriers and facilitators using the Consolidated Framework for Implementation Research.

**Methods:**

We conducted a qualitative case study across 3 academic medical centers from 2022 to 2024. Survey responses from clinical coordinators and principal investigators were coded within Consolidated Framework for Implementation Research domains. The intervention used automated text messages with links to patient education and interactive health assessments.

**Results:**

Patient engagement was limited at all sites. Pre-existing vaccination programs facilitated integration, whereas sites without established infrastructure faced greater difficulty. Institutional constraints, including institutional review board restrictions that excluded two-thirds of patients at one site, limited enrollment. Many patients distrusted unsolicited automated messages and perceived embedded links as phishing attempts. Weekly staff time varied substantially, and turnover disrupted implementation continuity. Despite limited platform engagement, the initiative increased provider attention to vaccination.

**Conclusion:**

Digital vaccine initiatives require more than technology deployment. Successful implementation should include early assessment of institutional capacity, patient-centered design addressing trust and communication preferences, baseline needs assessment, structured implementation support, and clear gastroenterology-primary care workflows. Hybrid approaches combining digital outreach with communication from known clinicians or nurses may be more effective than fully automated messaging for preventive care in inflammatory bowel disease.

## Introduction

### Background and Rationale

Inflammatory bowel disease (IBD), which includes Crohn's disease and ulcerative colitis, affects over 7 million people worldwide.^1^ IBD impacts diverse populations, including minoritized communities who may face significant healthcare disparities. Despite advances in treatment, racial, socioeconomic, and geographic disparities in IBD care remain substantial. Black, Hispanic, and lower-income populations experience higher hospitalization rates, decreased access to specialist care, and suboptimal medication adherence compared to their White and higher-income counterparts.^2^^,^^3^

Minoritized populations consistently receive less preventive care across healthcare settings, and this pattern extends to IBD management. Digital health interventions, including telemedicine, mobile health applications, and electronic health record (EHR)-integrated tools, have shown promise for enhancing patient engagement, promoting preventive health behaviors, and improving disease outcomes. For example, myIBDcoach has been associated with increased outpatient visit adherence,^4^ while the TELE-IBD program improved treatment adherence and provider-patient communication.^5^ Other IBD digital health and telemedicine interventions, including educational text messaging, patient-reported outcome platforms, population-based digital outreach, and telehealth coaching models, have similarly demonstrated feasibility for improving knowledge, monitoring, and access to care.^6^, ^7^, ^8^, ^9^, ^10^, ^11^ These tools may help bridge care gaps by expanding access to specialized care and improving disease management.^12^^,^^13^

IBD patients face unique barriers to vaccination delivery, including provider knowledge gaps about immunization recommendations, patient logistical challenges in managing multiple vaccines while accessing specialist care, and system-level constraints such as inability to stock vaccines due to cost and storage concerns.^14^ Vaccination represents a particularly important, yet challenging, domain for preventive care in IBD populations, and interventions to improve preventive care adherence in IBD often require system-level workflow support in addition to patient education.^15^

### Study Context and Objectives

We implemented a digital health intervention designed to increase compliance with vaccine recommendations among IBD patients across 3 academic medical centers serving diverse populations. The intervention, developed and deployed by Rx.Health/Commure Engage between 2022 and 2024, utilized automated text message outreach with embedded links to patient education and interactive health assessments. Implementation occurred during a period of evolving vaccine sentiment in the United States, when trust in both vaccination and digital health communications faced unprecedented challenges. We conducted systematic analysis using the Consolidated Framework for Implementation Research (CFIR)^16^ to understand what went wrong and why; these insights could prevent similar failures in future digital health initiatives.

Our retrospective application of CFIR is intentional and reflects a critical gap in digital health implementation. Too often, interventions are designed and launched without adequate implementation science assessment, leading to predictable failures that could have been avoided through upfront stakeholder engagement, needs assessment, and barrier identification. By transparently analyzing the barriers we encountered, we aim to demonstrate why implementation science must be engaged at project inception, not applied retroactively after interventions fail. This approach aligns with growing recognition within the IBD research community that successful digital health adoption requires rigorous upfront planning grounded in implementation science frameworks.

### Study Objectives

Using qualitative survey data gathered from site coordinators and principal investigators (PIs) at project conclusion, this study aims to:

1. Assess the feasibility and effectiveness of digital health interventions in improving vaccination rates, patient engagement, and health literacy among IBD patients.
2. Identify implementation barriers and facilitators using the CFIR framework, examining factors such as technology integration, institutional constraints, and provider engagement.
3. Compare site-specific challenges and successes, providing insights into how organizational and contextual factors influence digital health implementation.
4. Generate actionable recommendations for future digital health interventions in specialty care, emphasizing the critical importance of conducting implementation science assessments before intervention launch.

## Methods

### Study Design

This study employed a multisite qualitative case study design to examine the implementation of a digital health intervention aimed at improving vaccination uptake among patients with IBD. The research was conducted at 3 academic medical centers—designated as site A, site B, and site C—that participated in a national digital health pilot program coordinated by Rx.Health/Commure Engage (Figure).

**Figure:**
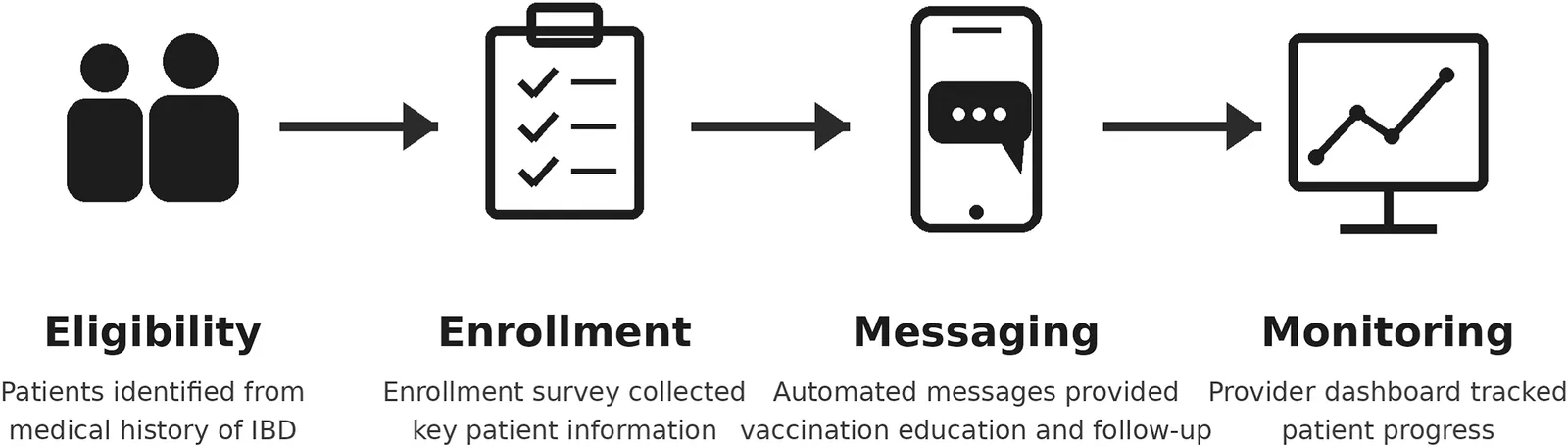
Digital health intervention workflow for vaccination. Eligible patients with inflammatory bowel disease were identified from the electronic health record, enrolled through a survey, contacted with automated vaccination education and follow-up messages, and monitored through a provider dashboard.

This study focused on how the intervention was operationalized within real-world healthcare settings. Using CFIR, we analyzed institutional, individual, and contextual factors that influenced implementation outcomes. Semi-structured surveys were administered to site leads, including clinical coordinators and PIs, to capture their experiences with deploying the intervention. Responses were coded across CFIR domains—Intervention Characteristics, Inner and Outer Setting, Characteristics of Individuals, and Implementation Process—to identify key barriers, facilitators, and cross-site variability.

### Study Setting and Participants

Each of the 3 study sites served distinct patient populations and had unique institutional structures that influenced implementation outcomes:

Site A: A major urban academic medical center with a well-established IBD program. This site primarily serves a racially and socioeconomically diverse patient population, including a significant proportion of Medicaid beneficiaries and underinsured patients.

Site B: A large safety-net hospital in the Bronx, New York, serving a predominantly Black and Hispanic population with a high proportion of Medicaid beneficiaries and uninsured patients, multiple medical comorbidities, and complex healthcare needs.

Site C: A regional academic health care center serving urban and rural patients, including a substantial Native American population and many Indian Health Service, Medicare, and Medicaid beneficiaries.

Because this evaluation analyzed implementation survey responses from site coordinators and PIs rather than patient-level clinical data, individual patient demographic characteristics were not available. Specifically, we did not collect age, sex, race/ethnicity, preferred language, socioeconomic indicators, insurance status at the individual level, baseline vaccination status, digital access, or formal measures of health literacy or medical mistrust. To provide context for cross-site differences, we summarized available site-level characteristics, including geographic setting, general population served, payer mix, existing vaccination infrastructure, and qualitative observations related to digital trust and health literacy (Table 1). These site-level descriptions should be interpreted as contextual information rather than patient-level demographic data.

**Table 1:** Site-Level Contextual Characteristics Across Participating Centers

Contextual characteristic	Site A	Site B	Site C
Geographic/clinical setting	Major urban academic medical center with established IBD program	Large Bronx safety-net hospital	Regional academic health care center serving urban and rural patients
Site-level population context	Racially and socioeconomically diverse population	Predominantly Black and Hispanic population with high medical complexity	Mixed urban/rural population with substantial Native American patient population
Payer/socioeconomic context	Significant proportion of medicaid beneficiaries and underinsured patients	High proportion of medicaid beneficiaries and uninsured patients	Indian Health Service, medicare, and medicaid beneficiaries represented
Existing vaccination infrastructure	Prior experience with vaccination work through IBD QORUS	No established patient vaccination program or dashboard before the project	No comparable pre-existing digital vaccine workflow; project prompted updates to clinic notes/smart phrases
Digital access/health literacy/trust	Coordinator perceived many patients as already engaged and health-literate	Not formally measured; implementation affected by safety-net setting, IRB constraints, and infrastructure limitations	Patients frequently questioned the legitimacy of text links, and some required more personal or simplified communication
Patient-level demographic data	Not collected	Not collected	Not collected

Participants across all 3 sites included gastroenterologists, nurse coordinators, and technology support staff who were actively involved in implementing the digital health intervention. Providers were recruited via purposeful sampling, ensuring representation across clinical, administrative, and technical roles at each site.

### Intervention Description

The digital vaccine intervention consisted of automated text messages sent to phones of IBD patients identified through EHR review. Messages contained embedded links directing patients to an online platform where they could:

• Review personalized vaccination recommendations based on their IBD medications
• Access educational materials about vaccine safety and importance
• Complete interactive health assessments
• Schedule vaccination appointments

The platform was designed to send automated reminders at regular intervals to patients who did not initially engage. Clinical coordinators at each site were responsible for identifying eligible patients, monitoring engagement metrics, and following up with patients who expressed interest or concerns.

### Data Collection

Data collection was conducted during the 6 months following the 36-month implementation period across all 3 sites (2022–2024). An online survey was conducted at each site with the clinical research coordinator (site A) or site PI (sites B and C) at the conclusion of the project. The survey questions were aligned with CFIR domains to explore both facilitators and barriers to implementation. Participants were asked to describe their experiences with digital health tools, the factors that influenced adoption, and the challenges they faced in integrating these tools into clinical workflows. Questions also addressed patient engagement and responses to digital health interventions.

### Data Analysis

All qualitative data were analyzed using a CFIR-guided thematic analysis approach. Survey responses were manually coded and tallied using Microsoft Excel according to CFIR's 5 major domains: Intervention Characteristics, Outer Setting, Inner Setting, Characteristics of Individuals, and Implementation Process. A deductive coding approach was employed to categorize data within these constructs, ensuring a systematic evaluation of implementation barriers and facilitators.

Following the initial coding, a thematic analysis was conducted to identify recurring patterns and site-specific variations. Responses were compared across participating clinical sites to determine common implementation challenges and contextual factors that influenced adoption. Special attention was given to distinguishing cross-cutting themes that emerged across multiple sites from those that were unique to specific settings. Coding and theme development were iterative and refined through team discussions to enhance credibility and consistency in CFIR domain assignment across sites.

## Results

### Overview of Implementation Outcomes

The digital vaccine initiative achieved limited patient engagement across all 3 sites, with implementation barriers substantially impeding adoption. While some sites successfully embedded vaccine awareness activities within their workflows, none achieved the anticipated level of direct patient engagement with the digital platform. The use of the CFIR enabled a structured analysis of facilitators and barriers to implementation across sites (Table 2).

**Table 2:** Qualitative Analysis: CFIR Domain Findings

CFIR domain	Assertion	Representative quote
Intervention characteristics	Sites with prior vaccination programs reported easier integration	“Yes, we have worked with IBD QORUS (CCF) on their vaccination initiative.” (site A)
Intervention characteristics	Sites without pre-existing vaccination frameworks struggled significantly	“There was no existing program for patients. Vaccine rates were not addressed prior to the project, there was no dashboard to show vaccine rates, charts may or may not have been checked to determine if a patient had their recommended vaccines.” (site B)
Intervention characteristics	Automated messaging was perceived negatively by patients and viewed with suspicion	“But in this day and age, people don't trust correspondence from people they don't know or are not expecting to hear from and definitely don't trust links. We received many calls/messages asking if the links were sent by us, and whether their HIPAA confidentiality is maintained.” (site C)
Outer setting	IRB constraints limited the scope of implementation at some sites	“2/3 of site visits were outside of the IRB approvals which limited the involvement of patients.” (site B)
Outer setting	Institutional limitations affected ability to implement certain features	“The weakness was the institutional lack of support and IRB not being responsive to the needs of providers, the lack of willingness of the local IRB which prohibited 2/3 of patient population from being enrolled. We were not able to bulk prescribe because of the system limitation at montefiore, ultimately an administrative issue.” (site B)
Outer setting	Technology infrastructure varied across sites, with some facing significant limitations	Site B could not utilize bulk prescribing features due to EHR system limitations; all sites required significant manual effort to extract EMR data for patient identification
Inner setting	Staff turnover affected implementation continuity	“Yes” [in response to staffing changes] (site A). “What we found a little challenging was the turnover in the leadership staff of the Rx.Health” and “current study coordinator was added after the implementation of the study.” (site C)
Inner setting	Time commitment varied significantly across sites	“4 [h]” (site A) vs “1–2 hours PI, 12 hours for coordinator” (site B) vs “5–6 hours” (site C) per wk
Characteristics of individuals	Training effectiveness and preparedness varied across sites	Sites A and C felt prepared to launch; site B did not feel prepared to launch
Characteristics of individuals	Patient digital literacy and trust levels significantly affected engagement	“Patients don't like answering the phone for people they do not know, and the ones that our nurse coordinator did talk to said they 'didn't trust clicking on links they get in a text message'.” (site C)
Characteristics of individuals	Patient perception of intrusion limited engagement	“Some patients felt the model was a little intrusive” and patients “were not pleased with the ongoing participation after choosing to opt-out.” (site C)
Process	Communication methods with patients significantly influenced success	“The primary weakness was the ease of use for patients (required too much time and effort and the constant pinging on contact information we provided were difficult).” (site A)
Process	Provider awareness increased as a result of the project, independent of patient engagement	“The positive aspect of this project was the act of enrolling or trying to enroll patients which actually resulted in patients getting vaccinated because they were thinking about it and I was thinking about it. Since the provider was thinking about vaccines because of the project, I made more vaccine recommendations as a result.” (site B)
Process	Quarterly check-ins between sites were valuable for implementation support	“The quarterly check-ins were most helpful when communication with other sites' coordinators, to get tips about how to engage with patients effectively when conducting via phone.” (site A)
Process	Sustainability concerns emerged due to limited perceived value relative to effort	“We felt that because of the limited response rate, the nurse coordinator, IBD team, and IBD nurse did not feel that the project was as valuable as it was thought to be.” (site C)
Process	Mismatch between intervention and population needs at some sites	“Our project has very engaged and interested patients - I think it is a valuable resource for smaller, rural, or less health-literate patient communities. However, given that our patients are often seeing our clinician after being familiar with their disease and are primarily highly educated and health-literate patients, we did not see the immediate and intense impact that this project may have been intended to demonstrate.” (site A)

CCF, Crohn’s & Colitis Foundation; EMR, electronic medical record; HIPAA, Health Insurance Portability and Accountability Act.

### Implementation of the Inflammatory Bowel Disease Vaccine Initiative Across Sites

Our analysis of survey responses from participating sites revealed several critical factors that influenced the implementation and effectiveness of the IBD vaccine initiative. By applying the CFIR, we identified key themes across 5 domains that shaped implementation outcomes.

### Intervention Characteristics: Usability and Adaptability

The feasibility of integrating the vaccine initiative into existing workflows varied across sites. Site A, which had prior experience with vaccination programs through IBD QORUS (Crohn’s & Colitis Foundation), reported more seamless integration of the new program into existing workflows.

Sites B and C, without pre-existing vaccine frameworks, faced some adaptation challenges. Site B reported that “there was no existing program for patients” and “vaccine rates were not addressed prior to the project.” This lack of baseline infrastructure meant these sites were simultaneously building vaccination awareness programs while attempting to implement a complex digital intervention—a dual burden that can be difficult to manage.

A critical challenge across all sites involved patient engagement with the platform itself. Beyond trust issues discussed below, site A identified “the ease of use for patients” as “the primary weakness” of the project, noting that it “required too much time and effort and the constant pinging on contact information we provided were difficult.” The multistep process of clicking links, navigating the portal, and completing assessments created friction that limited engagement.

### Outer Setting: Institutional and Policy Influences

Institutional support and regulatory requirements significantly impacted implementation, particularly at site B. Institutional review board (IRB) approval limitations meant that “2/3 of site visits were outside of the IRB approvals, which limited the involvement of patients.” This regulatory barrier fundamentally constrained the site's ability to implement the initiative, excluding the majority of their patient population before implementation even began. The site characterized this as “the institutional lack of support and IRB not being responsive to the needs of providers,” highlighting how regulatory processes designed for traditional research may not accommodate quality improvement initiatives or digital health interventions appropriately.

Technological infrastructure capabilities also varied between sites, affecting implementation feasibility. Site B was “not able to bulk prescribe because of the system limitation,” an EHR constraint that prevented efficient vaccine ordering even when patients expressed interest. All sites reported that identifying eligible patients required “significant labor hours and effort to manually extract electronic medical record data,” creating substantial workload burden for already-stretched clinical staff.

### Inner Setting: Organizational Support and Staffing

Staffing stability and time allocation emerged as critical factors affecting implementation. Weekly time commitments varied substantially across sites: 4 hours at site A, 13 to 14 combined hours at site B (1–2 hours for PI, 12 hours for coordinator), and 5–6 hours at site C. This variation reflects differences in patient population size, baseline infrastructure, and implementation challenges.

Both sites A and C reported staffing changes during the project period, potentially affecting continuity of implementation and institutional knowledge. Additionally, turnover within the Rx.Health/Commure Engage team itself created challenges. Site C noted, “What we found a little challenging was the turnover in the leadership staff of the Rx.Health,” and mentioned that their “current study coordinator was added after the implementation of the study.” These staffing changes—both at clinical sites and within the sponsoring organization—disrupted implementation momentum and required repeated knowledge transfer.

Positive workflow changes did emerge as an unintended benefit. Site C noted that “implementing the project led to updating our clinic notes/smart phrases,” especially with trainees, demonstrating how the implementation process itself prompted improvements in vaccination documentation practices that may outlast this particular intervention.

### Characteristics of Individuals: Provider and Patient Engagement

Provider readiness varied significantly across sites. When asked if they felt prepared to launch the project, sites A and C responded affirmatively, while site B did not. Despite feeling prepared, the sites achieved limited effective implementation, suggesting that perceived preparedness alone was insufficient without adequate assessment of institutional barriers, patient preferences, and infrastructure requirements.

Patient engagement challenges were consistently reported across all sites, particularly regarding trust in digital communications. Site C reported patients perceived messages as potential “phish attempts,” explaining that “people don't trust correspondence from people they don't know or are not expecting to hear from and definitely don't trust links.” Multiple patients called clinical coordinators asking “if the links were sent by us and whether their Health Insurance Portability and Accountability Act confidentiality is maintained,” demonstrating that even when messages came from legitimate healthcare sources, patients remained suspicious.

Site C further explained that “patients don't like answering the phone from people they do not know, and the ones that our nurse coordinator did talk to said they ‘didn't trust clicking on links they get in a text message’.” This fundamental distrust of digital outreach—particularly automated links—created a significant barrier to engagement for many patients. Some patients found the approach “intrusive,” and site A noted that “patients were not pleased with the ongoing participation after choosing to opt out,” suggesting that the automated reminder system sometimes failed to respect patient preferences.

### Process: Communication and Sustainability Challenges

Communication methods significantly influenced implementation experiences. Quarterly check-ins between sites were highlighted as particularly valuable, with site A noting these were “most helpful when communication with other sites' coordinators to get tips about how to engage with patients effectively when conducting via phone.” This learning opportunity across sites provided a form of implementation support, though communication with patients remained challenging throughout implementation. The basic engagement mechanism—“having the patients click on the link”—was identified as the primary barrier, as this click was “considered the ‘trigger’ for the project.” When patients refused to click links or ignored messages for the reasons outlined above, the entire intervention pathway was simply blocked.

Despite implementation challenges and limited patient engagement with the digital platform, an important positive outcome emerged: increased provider awareness of vaccination needs. Site B noted, “The positive aspect of this project was the act of enrolling or trying to enroll patients, which actually resulted in patients getting vaccinated because they were thinking about it and I was thinking about it. Since the provider was thinking about vaccines because of the project, I made more vaccine recommendations as a result.” Site C echoed this, noting the project “reminded our ancillary staff and patients about the importance of vaccines.” This “provider awareness effect” represents a meaningful benefit independent of the digital platform itself.

Sustainability concerns were prominent across all sites, with uncertainty about continuing the initiative without ongoing support. Site C stated, “We felt that because of the limited response rate, the nurse coordinator, the IBD team, and the IBD nurse did not feel that the project was as valuable as it was thought to be.” This perception that effort outweighed benefit raises fundamental questions about intervention design and the importance of conducting needs assessment and pilot testing before full implementation.

### Impact on Minoritized Populations

When asked about the potential of this model to improve vaccine rates for minoritized IBD patients, responses were mixed and reflected some of the implementation challenges described above. Site A believed the model could be effective for “smaller, rural, or less health-literate communities.” However, site C, which does serve rural and diverse populations, expressed reservations: “Some disadvantaged patient population needs more of an in-person or more simplified communication method. Some patients felt the model was a little intrusive.”

This highlights a critical implementation science lesson: interventions designed without direct input from target populations may fail to address their actual barriers and preferences. The automated digital approach may have inadvertently created new barriers for the very populations it aimed to serve.

## Discussion

We examined the implementation of a digital vaccine initiative for IBD patients across 3 major healthcare sites, revealing critical insights into the factors that influence successful adoption of digital health interventions for preventive care in specialty populations. Using the CFIR, we identified key facilitators and barriers that shaped implementation outcomes. Our findings demonstrate that digital health interventions can have limited impact when deployed without adequate preparation, institutional support, provider and team engagement, and patient trust in digital communication methods.

This study adds 3 key contributions: (1) documentation of how institutional and regulatory constraints can limit digital health interventions, (2) demonstration that patient distrust of automated digital communications can undermine interventions even when access and technology are provided, and (3) characterization of a “provider awareness effect,” whereby digital health initiatives can improve preventive care through changes in provider behavior even when direct patient engagement with platforms remains limited.

### Principal Findings

The implementation success was highest at the site with the least need for intervention. Site A, which had prior vaccination program experience through IBD QORUS (Crohn’s & Colitis Foundation), reported smoother integration to patients who were already receiving comprehensive care. This represents a flaw in implementation strategy: the intervention was deployed without adequate needs assessment to ensure it matched population characteristics and potential gaps in care. This mismatch underscores the critical importance of engaging stakeholders before intervention rollout.

Institutional support emerged as a critical determinant of implementation success. Site B faced IRB constraints that excluded two-thirds of eligible patients from participation. The site characterized this as “IRB not being responsive to the needs of providers,”. This may raise the concern that quality improvement initiatives using digital health tools occupy a “gray zone” in the regulatory space between research and clinical care. This suggests the need for frameworks that more appropriately assess digital health quality improvement initiatives.

Patient distrust of automated digital communication emerged as likely the most significant barrier, cutting across all 3 sites regardless of their different patient populations and institutional contexts. Patients perceived automated text messages with embedded links as potential “phish attempts,” called coordinators to verify message legitimacy, and expressed concerns about “Health Insurance Portability and Accountability Act confidentiality.” This finding is particularly significant given the timing of implementation (2022–2024), a period when vaccine hesitancy intersected with increasing concerns about digital privacy and increasing digital and phishing scams.

The distrust was not only about vaccination but also about the communication method itself. As site C noted, “people don't trust correspondence from people they don't know or are not expecting to hear from and definitely don't trust links.” This represents a significant challenge for automated digital outreach: even when messages come from healthcare sources, patients cannot easily distinguish them from malicious communications. Some patients found the approach “intrusive,” particularly when automated reminders continued after opting out.

This trust barrier may be more prominent in minoritized populations who may have experienced, or are more vulnerable to digital scams. Site C's observation that “some disadvantaged patient population needs more of an in-person or more simplified communication method” suggests that automated digital approaches may inadvertently increase healthcare disparities rather than address them. Without patient-centered design that addresses these concerns, digital interventions risk creating new barriers for the populations they aim to serve.

An important, yet unexpected, finding was the “provider awareness effect.” Even though patients had limited engagement with the digital platform, the process of identifying eligible patients and attempting to enroll them increased provider attention to vaccination needs. Site B reported that “the act of enrolling or trying to enroll patients actually resulted in patients getting vaccinated” because “the provider was thinking about vaccines because of the project.” This suggests that interventions can improve care quality by changing provider behavior, independent of whether patients adopt the technology itself.

Our findings also suggest that a purely patient-directed digital vaccination intervention may be insufficient for patients with IBD. Although patient education and digital outreach can improve awareness, vaccination decisions in IBD are often clinically nuanced because recommendations depend on immune-modifying therapy, vaccine type, timing relative to immunosuppression, and coordination between gastroenterology and primary care. The 2025 American College of Gastroenterology preventive care guideline reinforces the need for age-appropriate vaccination before initiation of immune-modifying therapy when possible and avoidance of live vaccines in immunosuppressed patients.^17^ However, translating these recommendations into practice remains challenging. Prior studies have demonstrated knowledge gaps among gastroenterologists and primary care professionals, with gastroenterologists often viewing vaccination as the responsibility of primary care and primary care clinicians reporting unfamiliarity or discomfort with IBD-specific vaccine recommendations.^18^, ^19^, ^20^ In addition, patient-facing education alone may not reliably change vaccine behavior; Project PREVENT demonstrated the feasibility of web-based preventive care education in IBD, while broader intervention studies suggest that improvements in provider knowledge, workflow support, and vaccine availability may be needed to translate awareness into uptake.^21^^,^^22^ These findings align with our observation that the intervention itself increased provider attention to vaccination needs, even when direct patient engagement with the digital platform was limited. Future programs should therefore pair patient-facing digital tools with provider-facing education, EHR-based decision support, standardized vaccine checklists, and explicit workflows defining shared responsibility between gastroenterology and primary care.

The role of IBD nurses deserves particular emphasis. Although staff at site C felt that the project was less valuable than anticipated because of low response rates and high effort, this should be interpreted as a limitation of intervention fit and implementation burden rather than as evidence against nurse involvement. IBD nurses are often trusted points of contact for patients and can provide practical counseling about vaccine safety, timing, and logistics. Studies have suggested that patients expect IBD nurses to contribute to vaccine education, while on-site vaccine availability in IBD clinics, including nurse-supported workflows, can improve vaccine discussion and completion for selected vaccines.^23^^,^^24^ Nurse-led or nurse-supported outreach may be especially important for underserved populations, where trust, continuity, language access, and culturally responsive communication may determine whether patients accept preventive care recommendations. Future digital vaccine programs should include IBD nurses early in intervention design, provide protected time and standardized scripts, and use nurses as part of a hybrid model that introduces digital tools through known members of the care team rather than relying solely on automated messages from unfamiliar sources.

### Barriers to Implementation and Lessons Learned

Our experience reveals multiple levels at which digital health interventions can have limited success:

1. Misalignment with population need: The intervention achieved the easiest integration at the site that might have needed it least, while facing challenges at sites serving populations with greater barriers to care. This suggests that pilot testing before full deployment might have addressed this issue.
2. Inadequate patient-centered design: The intervention was designed without formative research to understand patient preferences, trust levels, and barriers. This might have been mitigated if we engaged patients in design and learned about distrust of automated links and preferences for provider-initiated communication methods.
3. Regulatory barriers: Traditional IRB processes excluded two-thirds of patients at one site, suggesting that regulatory frameworks have not adapted to accommodate digital health quality improvement initiatives appropriately.
4. Infrastructure limitations: Sites faced varying technological constraints, from inability to bulk-prescribe vaccines to labor-intensive manual data extraction. These infrastructure barriers created additional burdens for clinical staff.
5. Unclear gastroenterology-primary care ownership: The intervention did not include a structured shared-care pathway between gastroenterologists and primary care clinicians. This is a critical limitation for IBD vaccination because recommendations may depend on immunosuppressive therapy, anticipated treatment escalation, and avoidance of live vaccines during immunosuppression. Without explicit ownership of vaccine assessment, counseling, ordering, administration, and documentation, responsibility can diffuse across clinical teams, leaving patients uncertain about whose recommendation to follow and providers uncertain about who should act.
6. Communication method mismatch: Automated text messages with embedded links proved fundamentally misaligned with patient trust levels and preferences, particularly during a period of increased vaccine skepticism and digital fraud concerns.
7. Staffing instability: Turnover at both clinical sites and within the sponsoring organization (Rx.Health/Commure Engage) disrupted implementation continuity and required repeated knowledge transfer.

### Implications for Future Implementation

Based on our experience, we propose a framework for implementing future digital preventive care interventions in specialty settings:

### Before Launch: Essential Preparation

1. Conduct needs assessment: Systematically evaluate which patient populations face the greatest barriers to preventive care and whether digital interventions address their actual needs vs assumed needs.
2. Engage patients in study design: Conduct formative research with patients from target populations to understand their preferences, trust levels, barriers, and communication preferences before designing interventions.
3. Assess institutional capacity: Evaluate IRB requirements, technological infrastructure, staffing availability, and existing workflows before committing to implementation.
4. Ensure appropriate regulatory approval: Work with IRBs to establish frameworks appropriate for digital health quality improvement that don't inadvertently exclude target populations.
5. Pilot test at small scale: Test interventions with small patient cohorts, gather feedback, and iterate on design before large-scale deployment.

Future programs should also assess provider knowledge and readiness before launch. This may include brief gastroenterology and primary care education on IBD-specific vaccine recommendations, standardized vaccine checklists embedded in clinic workflows, and EHR-based prompts that identify vaccine needs before visits. Importantly, workflows should specify whether the gastroenterology clinic will recommend, order, administer, or refer for vaccination, and how completion will be documented and communicated back to the care team.

### During Implementation: Critical Supports

1. Provide structured communication: Establish regular check-ins between sites for peer learning and real-time problem-solving, as demonstrated by our quarterly calls, which were well received by staff.
2. Monitor engagement metrics: Track not just patient engagement but also staff time burden, provider behavior changes, and unintended consequences.
3. Maintain flexibility: Be prepared to modify communication strategies and workflows and pivot approaches based on ongoing experience with the project.
4. Ensure staffing stability: Minimize turnover and ensure adequate time allocation for implementation activities.

### Communication Strategy: Hybrid Provider- and Nurse-Supported Approaches

Our findings suggest that future preventive care interventions should strongly consider hybrid approaches that combine digital tools with direct communication from known members of the care team rather than relying solely on automated outreach. Options include:

• Provider-initiated conversations about vaccination, supplemented by digital resources
• Handoffs where providers introduce digital tools in clinical encounters
• Text messages that come from named, known providers rather than generic automated systems
• In-person or telephone outreach for initial engagement, with digital tools for follow-up

These hybrid approaches may be more patient-centric and resource-intensive than fully automated systems, but appear more likely to succeed given patient trust concerns and preferences for human interaction, particularly for vulnerable populations.

These approaches should include nurses and coordinators only with adequate training, protected time, and clear escalation pathways so that vaccine navigation improves patient trust without adding unsustainable burden to clinic staff.

### Strengths and Limitations

This study offers a structured examination of digital health implementation across diverse clinical settings, providing transparent analysis of implementation failure and practical lessons for future efforts. The use of CFIR as an analytical framework enables systematic categorization of implementation factors, while our multisite design reveals how contextual factors can influence implementation outcomes.

However, several important limitations warrant consideration. First, although the study included 3 academic medical centers, the sites differed substantially in social context, patient populations, payer mix, existing vaccination infrastructure, regulatory processes, EHR capabilities, and staffing resources. These differences likely influenced implementation outcomes and limit direct cross-site comparison.

Second, individual patient-level demographics were not collected because this evaluation analyzed qualitative implementation survey data from site coordinators and PIs, and no patient-specific data were used. As a result, we could not evaluate whether engagement differed by age, sex, race/ethnicity, preferred language, insurance status, socioeconomic status, baseline vaccination status, digital access, health literacy, or medical mistrust. Future studies should prospectively collect these variables and incorporate validated measures of digital access, preferred communication modality, health literacy, and trust in healthcare systems to better explain site-level differences in uptake.

Third, our data were derived primarily from written survey responses rather than in-depth interviews with patients, nurses, gastroenterologists, primary care clinicians, or implementation staff. Therefore, we could not fully explore patient decision-making, provider knowledge gaps, nurse perspectives, or the reasons patients declined or ignored outreach.

Fourth, the intervention did not include a structured mechanism for coordination between gastroenterologists and primary care physicians. This is particularly important in IBD, where vaccine recommendations may depend on immunosuppressive therapy and timing considerations, yet responsibility for vaccination is often distributed ambiguously between specialty and primary care. Finally, the intervention was implemented during 2022-2024, a unique period when vaccine hesitancy, concerns about privacy, and distrust of unsolicited digital links were especially salient. Findings may not fully generalize to other time periods or other preventive care interventions beyond vaccination.

## Conclusion

Our study demonstrates that implementing digital vaccine initiatives for IBD patients involves navigating complex interactions between institutional factors, technological capabilities, provider engagement, and patient trust. While digital tools offer promising opportunities to improve preventive care for IBD populations, their effectiveness depends on thoughtful implementation strategies that address barriers at multiple levels.

Future digital health initiatives should engage implementation scientists from project inception to conduct thorough needs assessment, engage patients from target populations in design, pilot test at a small scale, and build in approaches for real-time adaptation.

For vaccination and other preventive care interventions in specialty populations, hybrid approaches combining digital outreach with direct provider, primary care, and nurse-supported communication may be more effective than fully automated systems, particularly for minoritized populations who face barriers to care and may have justifiable reasons to distrust automated digital communications. Without this upfront investment in implementation science, even well-intentioned interventions risk creating new barriers for the populations they aim to serve.

## Declaration of Generative AI and AI-Assisted Technologies in the Writing Process

During the preparation of this work, the authors used Claude.ai and PerfectIT for initial draft assistance with manuscript language and structure, grammar and style refinement, and reference formatting. After using this tool/service, the authors reviewed and edited the content as needed and take full responsibility for the content of the published article.
